# Professionalism-training in undergraduate medical education in a multi-cultural, multi-ethnic setting in the Gulf Region: an exploration of reflective essays

**DOI:** 10.1186/s12909-024-05103-z

**Published:** 2024-02-06

**Authors:** Rasha Buhumaid, Farah Otaki, Katarzyna Czabanowska, Adrian Stanley, Mutairu Ezimokhai, Lisa Jackson, Samuel B. Ho

**Affiliations:** 1 College of Medicine, Mohammed Bin Rashid University of Medicine and Health Sciences, Dubai Health, Dubai, United Arab Emirates; 2 Strategy and Institutional Excellence, Mohammed Bin Rashid University of Medicine and Health Sciences, Dubai Health, Dubai, United Arab Emirates; 3 Department of Health Services Research, Care and Public Health Research Institute (CAPHRI), Faculty of Health, Medicine, and Life Sciences (FHML), Maastricht University, Maastricht, The Netherlands; 4 Department of International Health, Care and Public Health Research Institute (CAPHRI), Faculty of Health, Medicine, and Life Sciences (FHML), Maastricht University, Maastricht, The Netherlands; 5 Department of Health Policy Management, Faculty of Health Care, Institute of Public Health, Jagiellonian University, Kraków, Poland

**Keywords:** Professionalism, Undergraduate medical education, Gulf Region, United Arab Emirates, Adult learning theory, Kolb’s experiential learning theory, Situated Learning Theory, Self-directed learning, Qualitative research

## Abstract

**Background:**

Despite the established need to prioritize professionalism-training in developing future physicians, very few medical programs in the Gulf Region embed in their curricula discrete contextualized courses aimed at developing the corresponding competencies, while fostering self-directed learning. This study aims at exploring the perception of undergraduate medical students in a multi-cultural, multi-ethnic setting regarding their understanding of, and personal experience with professionalism through their engagement with the content of an innovative curriculum-based professionalism course, offered at a Medical School in Dubai, United Arab Emirates.

**Methods:**

The study used a qualitative phenomenological research design. Out of 33 students, 29 students had submitted reflective essays. The content of these essays was inductively analyzed following a six-step framework for conducting thematic analysis. The framework’s steps include familiarizing oneself with the data, generating initial codes, searching for themes, reviewing themes, defining and naming themes, and producing the report.

**Findings:**

The inductive qualitative analysis generated the *Professionalism Learning Journey* model. This conceptual model includes four interconnected themes: *Awareness, Acknowledgement, Realization, and Application.* The generated model depicts the trajectory that the learners appear to experience while they are engaging with the content of the course.

**Conclusion:**

Integrating a professionalism-training course into an undergraduate medical curriculum is likely to be positively appraised by the learners. It raises their awareness, enables them to value the subject matter and the sophistication of its application, and empowers them to put into practice the taught principles, on an individual basis and collectively. This is especially true when the course is entrenched in constructivism experiential learning theory and designed to foster self-directed learning. The introduced conceptual model, in conjunction with the innovative professionalism-training course curriculum, can serve as a template for other competencies and other schools.

**Supplementary Information:**

The online version contains supplementary material available at 10.1186/s12909-024-05103-z.

## Introduction

The literature on professionalism is extensive. Professionalism can be defined as “the conduct, aims, or qualities that characterize or mark a profession or a professional person” [1]. Medical professionalism is a fundamental competency in health professions’ education. It can be considered as the values, behaviors, and attitudes that foster professional relationships, public trust, and patient safety [2]. Defining medical professionalism is not a straightforward task [3], since it is a multidimensional, continuously evolving, socio-culturally informed construct that influences many aspects of care including but not necessarily limited to physician-patient relationships, patient satisfaction, and outcomes of care [4]. Moreover, existing definitions of what constitutes “professionalism” in the medical context vary. Concepts of professionalism seem to differ depending on culture and geographic location [5, 6]. International students learning and working with a standardized curriculum but out of their usual context require a tailored approach to their learning as they may demonstrate very different attitudes initially around concepts of professionalism [7]. Furthermore, there appears to be a generational gap in the understanding of professionalism, such as earlier concepts of detachment, paternalism, and restricted communication, versus newer concepts of empathy, emotional engagement, open communication, and patient autonomy [6].

Medical professionalism is a worldwide concern. Patients in every context expect physicians to be professional, and most medical organizations and societies specifically outline principles of professionalism they expect in their practitioners. In fact, medical professionalism is identified as a core competency in CanMEDS framework by the Royal College of Physicians and Surgeons of Canada [8] and by the Accreditation Council for Graduate Medical Education (ACGME) in the United States (US) [9]. In the United Kingdom, the General Medical Council (GMC) professional standards [10] rely on a process of annual appraisal in *Trust and professionalism*, along with three other domains: *Knowledge, skills and development*; *Patients, partnerships and communication*; and *Colleagues, culture and safety* [10].

Multiple studies have shown that professionalism is associated with improved medical outcomes, whereas “unprofessional” behavior is associated with adverse medical outcomes [11]. Despite medical professionalism’s established significance, there seems to be little consistency in how professionalism is taught to medical students [4, 12]. The structure of professionalism training in medical education is not unified and there is heterogeneity in the models of delivering the training. The sophistication of the subject makes teaching and assessing professionalism in medical education more challenging compared to teaching and assessing other competencies (e.g., medical knowledge or clinical skills) [12].

Traditionally, medical professionalism was developed through the unscripted hidden curriculum. This was heavily reliant on the learners’ observation of the faculty role modeling and on their socialization with the members of the clinical team. Interestingly, a lot of doctors believe that they are “professional” and that teaching professionalism is intuitive [13]. While students and residents tend to have positive views of professionalism-training, it is not unlikely for them to report observing what they consider as “unprofessional behaviors” in peers and in faculty [14]. Students, in a study conducted in the UK, reported confusion about what constitutes professionalism. They also reported concerns around how the professional identity can contribute to sense of a split in the persona, and even an unwelcome ‘sacrifice’ of sorts [15]. In a study exploring the perceptions in relation to the importance of structured professionalism education in medical training in Iran, trainees felt particularly unprepared for interprofessional teamwork in practice. They considered structured teaching in ethics and professionalism to be important, and only 1/5th considered role modelling as the sole method for learning professionalism [16]. When societies and the medical profession itself were reasonably homogeneous, values were shared and could be transferred effectively through role modelling. The increasing complexity of the ethical dilemmas faced by contemporary doctors, sophistication of the practice of medicine, and the diversity of the medical profession and of society makes transferring of professional values through role modeling insufficient [5, 17].

Nowadays there is commitment to advance professionalism-training and therefore most medical schools have formal activities that are incorporated in the curriculum [18]. It is established that professionalism needs to be explicitly taught and effectively assessed. Many faculty, however, do not possess the needed competencies to teach in this area and require faculty development [13, 19]. The importance of teaching and assessing professionalism for medical students has placed significant demands on medicine’s education institutions. A total of 204 medical students in Pakistan from years 1, 3, and 5 of the MBBS course were surveyed by using a scenario-based professionalism questionnaire and the performance of the students assessed. The results indicate a need to do more to improve students ‘professionalism knowledge, attitudes, and behaviours’ throughout the MBBS programme, and prevent declines in attitudes and behaviour throughout the training [20]. Similarly, conclusions of a systematic curriculum review in Wales, UK, which included a student survey around professionalism and ethics, suggested that students are more likely to find this type of teaching meaningful and applicable if it is integrated throughout the course, rather than just as an add-on [21].

A systemic scoping review, that investigated means by which professionalism is taught in medical schools, identified 4 themes [4]. These themes included the definition of professionalism, and the approaches, content, and the barriers and enablers to teaching professionalism. This review of the literature emphasized the importance of having a viable definition of professionalism with clear milestones for developing professionalism among medical students. This is expected to guide assessments, and clarify expectations, and roles and responsibilities of learners, while ensuring appropriate learning support along the professionalism-training process. The Association for Medical Education Europe (AMEE) developed an evidence-driven guide to integrating professionalism into the curriculum, highlighting four elements: (i) agreeing on an institutional definition for professionalism, (ii) structuring the curriculum to integrate professionalism learning across all years, (iii) anchoring the learning and teaching initiative in established theories of education, (iv) fostering the impact of the formal, informal, and hidden curricula, and (v) effectively assessing the learning [22]. It would be worthwhile to consider purposefully developing other complementary skills such as self-directed learning [23, 24] that would maximize the medical students’ contextualized professionalism learning experience. Also, since the learners need to continue developing their professional identity for the rest of their medical career, fostering self-directed learning as part of the formal professionalism-training becomes of great relevance. This requires for the design of professionalism learning and teaching activities to be anchored in educational theories, as recommended by the evidence-driven AMEE guide [22]. For example, Kolb’s learning cycle [25], as an experiential learning theory offers a useful theoretical framework for developing professionalism. To enable successful ‘reflection on action’, concrete experiences must be embedded in the curriculum. This is why complementing Kolb’s learning cycle with situated learning theory, which highlights that students learn through guidance in a structured learning environment, is important. Situated Learning Theory also emphasizes the practical reality that students observe and learn from expert role models. They learn through limited practice in the presence of a professional role model. As the learners become more experienced, they move closer to the center of institutional practice [22].

The teaching strategies reported upon in the literature include lectures, case studies, role modeling, mentoring, small-group discussions, reflective practice, and technological tools. Some programs introduced creative methods of conducting professionalism-training, such as the utilization of theater-based approach to teach first year medical and dental students at the University of Alberta [26]. Another innovative initiative reported in the literature was around developing the Medical Education e-professionalism framework (MEeP), which is meant to support healthcare professionals to cope with the challenges of medical professionalism in the digital realm. The framework reveals the interconnections between three aspects: values (adherence), behaviors (accountability), and identity (empathy and sensitivity), along with highlighting the competencies corresponding to each of these aspects [27]. Furthermore, interprofessional education can play an integral role in developing professionalism, along with teamwork, among healthcare professionals. The focus can be on attributes shared by all healthcare professionals, which will more adequately prepare students for working in healthcare teams. From this perspective, interprofessional education can provide appropriate methods to learn inter-professionalism, and this is expected to ultimately contribute to overcoming uni-professional exclusivity [28, 29].

The Gulf Region is on a par with the global acknowledgment of professionalism as a key element in medical education. For example, professionalism is highlighted as a fundamental aspect in medical practice in both the SaudiMEDs competency framework in the Kingdom of Saudi Arabia [30], and the more recently developed and endorsed EmiratesMEDs framework in the United Arab Emirates (UAE) [31]. To enable abiding by such frameworks, there needs to be a clear, contextual definition and understanding of professionalism. Adopting a definition of medical professionalism that proved viable in the Western world may not be fully applicable in the Gulf Region [32–35]. Accordingly, there has been several attempts of conceptualizing professionalism within the context of the Arab region. For example, one of the attempts, reported upon in the literature, revolves around a Delphi study which led to a consensus around the basic attributes of medical professionalism in the Arabian context. These attributes were all fitted into four themes: relating to self, to tasks, to others, and to God [36]. A more recent consensus conference of medical educators and senior leaders led to a more detailed articulation of the most important aspects of medical professionalism. This initiative considered the values, behaviors, and attitudes applicable to health professionals practicing in the Gulf Region. The content of the resultant document largely overlaps with Western concepts such as those highlighted in the American Board of Internal Medicine (ABIM) Physician Charter on Medical Professionalism, along with including crucial values unique to the Gulf Region [37]. Regional medical educators will need to find ways to operationalize those agreed upon contextual conceptualizations of professionalism. In other words, having a definition does not suffice in terms of developing the desired medical professionalism competencies. Efforts need to be directed towards translating those definitions into applicable learning and teaching interventions. This calls for the deployment of established theories of education to guide the designing of such interventions. Moreover, these learning and teaching initiatives need to begin with socio-culturally appropriate training in clinical competence, humanistic qualities, and reflective capacity. Core medical professionalism values can be shared but the diverse, ever-changing societal values inevitably influence institutional understanding and hence the identification of the desired educational outcomes.

Despite the established need to prioritize professionalism-training in developing future physicians, very few medical programs in the Gulf Region embed in their curricula discrete contextualized courses aimed at developing the corresponding competencies [35]. There is some research conducted in the region around medical professionalism [35, 38, 39], most of which investigate the perception of stakeholders (primarily the learners) about the concept of professionalism [35, 40]. Very few describe the planning, implementation, and evaluation of a relevant learning and teaching intervention [29]. None of the interventions reported upon are entrenched in a contextualized conceptualization of medical professionalism and are in alignment with established theories of education. Accordingly, the overall purpose of the study is to explore the perception of undergraduate medical students in a multi-cultural, multi-ethnic setting regarding their understanding of, and personal experience with professionalism through their engagement with the content of an innovative curriculum-based professionalism-training course, offered at a Medical School in Dubai, United Arab Emirates. Therefore, the current study addresses the following research question:

- How is professionalism understood by undergraduate medical students (trained in professionalism), and how do they perceive it in relation to their clinical placements?

## Methods

### Context of the study

The current study was conducted in the College of Medicine (CoM) at Mohammed Bin Rashid University of Medicine and Health Sciences (MBRU) in Dubai, United Arab Emirates. The CoM was established in 2016 with a six-year full-time Bachelor of Medicine and Bachelor of Surgery program (MBBS). The course of study is divided into three phases. Phase 1 and Phase 2 consist of basic sciences and organ system courses (years 1–3), followed by Phase 3 consisting of clinical placements (years 4–6, with year 6 following an apprenticeship model) [41]. The respective MBBS program fosters self-directed learning and students are encouraged throughout their learning journey to reflect on their experiences.

### Description of the professionalism-training course

The professionalism-training course was designed at the inception of the clinical placements in 2018. It was conceived as one of several ‘longitudinal curricular themes’ that would be delivered alongside the clinical placements. Its development was initiated by a workshop which focused its discussion on the how best to develop professionalism among undergraduate medical students during their clinical training. The workshop was led by the chair of the clinical sciences department (S.B.H.) and its membership was multi-disciplinary and included college faculty members and other staff at the university. To set the scene, S.B.H. presented a thorough review of the professionalism curricula in medical schools, worldwide, and a literature review of academic papers that focused on professionalism courses and competencies among medical students. The main workshop activity was a focus group session, whereby participants discussed and in turn agreed on the content (including but not limited to priority professionalism concepts for medical students) and the means of training delivery. It was agreed that the training will be curricular, structured longitudinally across year 4, built on existing western curricula, and contextualized to match intricacies of the Arab culture. There was emphasis on a secular consensus statement that encompasses culture and values relevant to professionalism for the UAE and the Arab region [37].

**Fig. 1:**
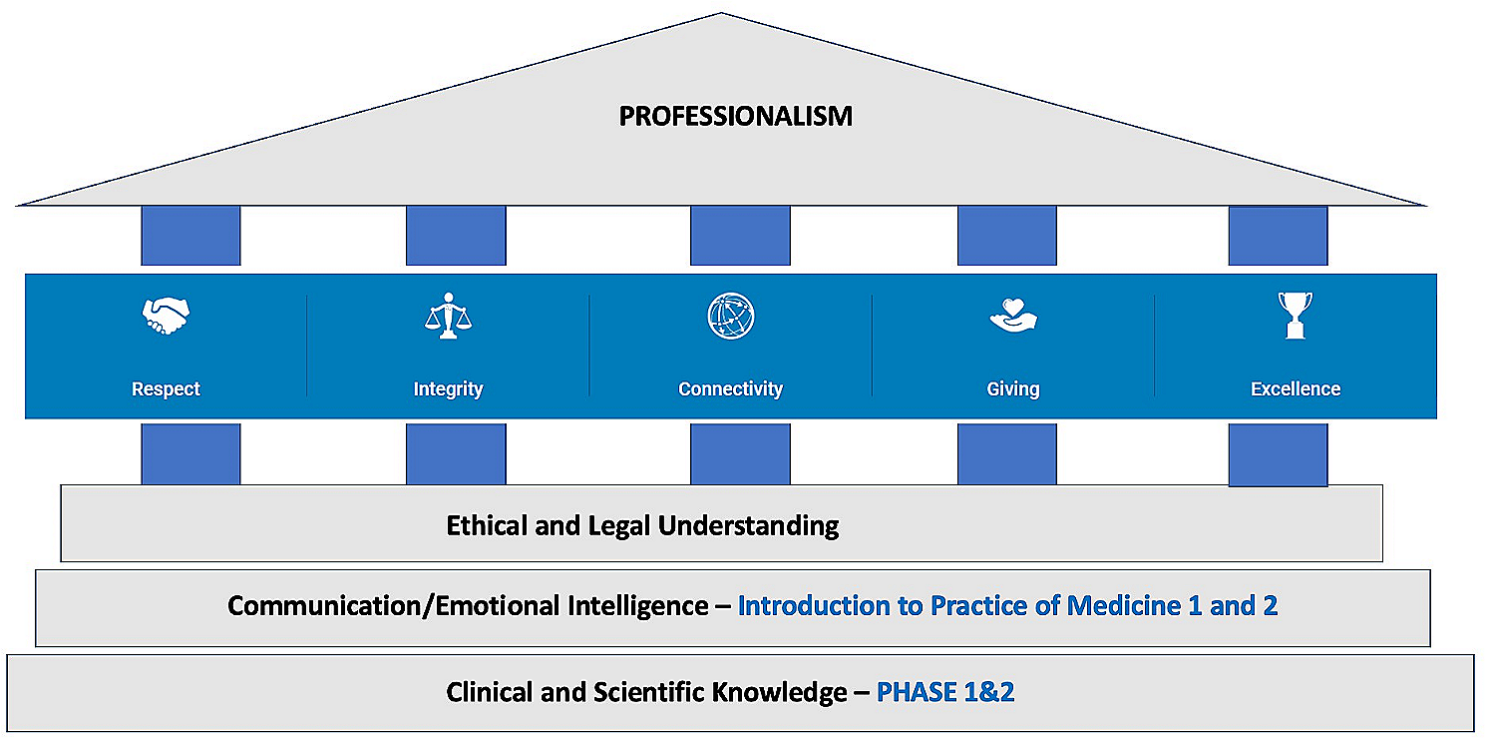
Basis of the professionalism course under investigation (institutional values and MBBS courses)

A faculty member (R.B.) was assigned the responsibility of leading the professionalism course’s development, implementation, and evaluation. A course team was formed, and individual faculty members were invited to contribute to the delivery of segments of the course. The course curriculum (Fig. 1) was built upon foundational courses in the MBBS curriculum in addition to values of the University [42]. The curriculum was designed to emphasize the MBRU values of *Respect, Integrity, Connectivity, Giving, and Excellence*, and built on the foundational courses in ethics and history of medicine that are included in Phases 1 and 2 of the MBBS program.

**Fig. 2:**
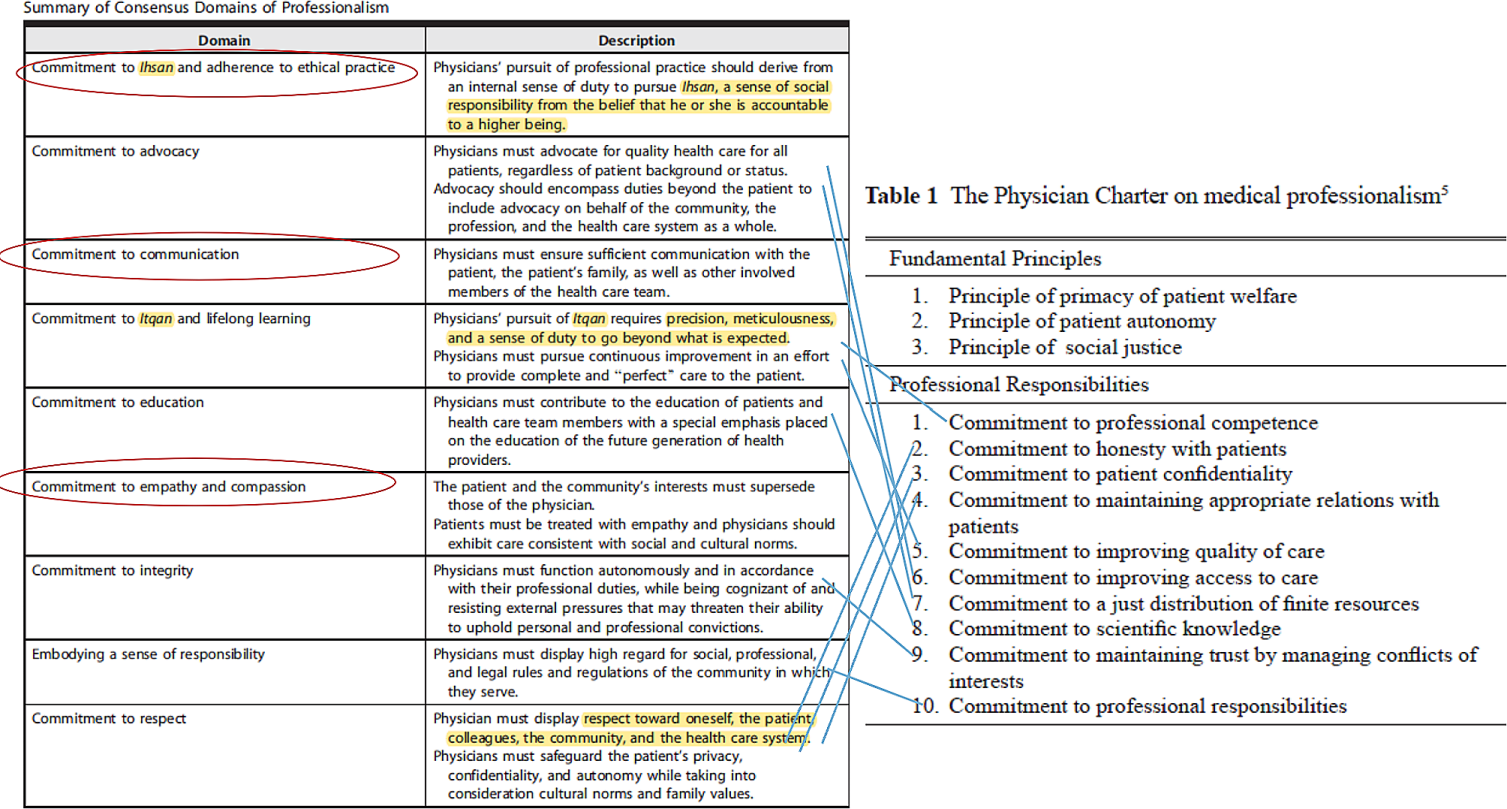
Comparison of the consensus domains of professionalism (this figure is composed of a modified consolidation of two illustrations- right extracted from ABIM physician charter on medical professionalism and left from abovementioned secular consensus statement)

The professionalism concepts that were selected based on the consensus statement [37] largely overlap with the American Board of Internal Medicine (ABIM) Physician Charter on Medical Professionalism that has been endorsed by over 90 medical specialties (Fig. 2) [43, 44].

In Year 4, students are allocated to their clinical placements for 4 days per week, with one day allocated to classroom tutorials. The latter is split equally between specialty-specific teaching relevant to the clinical placement, and the ‘longitudinal curricular themes’ which included the professionalism course.

The overarching aim of the 9-hour professionalism-training course is to raise awareness about issues related to professionalism that the students may encounter in the clinical workplace, and to provide them with the tools for understanding and developing professionalism skills (Supplementary Material). Within the context of this course, “medical professionalism” signifies a set of values, behaviors, and relationships that underpins the trust the public has in doctors [45]. The first session was a generic, stimulating introduction to medical professionalism, and subsequent sessions were focused on a commitment to the following concepts:

- Ihsan and adherence to ethical practice.
- Advocacy and sense of responsibility.
- Respect and communication.
- Itqan and lifelong learning.
- Teaching.
- Empathy and compassion.
- Integrity.

Each of the concepts were delivered over an hour, across consecutive weeks. The exception was commitment to respect and communication, which spanned two hours.

The professionalism-training course was designed for self-directed learning, and intrinsically motivated adult learners, based on the constructivism theory of experiential education [46]. Thus, the course enabled students to identify gaps in their own understanding, and to address these gaps through engagement in active learning, and through exercising analogical reasoning in learning and practice. The course was also aligned with Kolb’s experiential learning theory, reflection was fostered through embeddedness in the clinical environment (i.e., situational conditions), and the supervision of and feedback from senior clinician instructors who are experts in the subject matter. Within this context, from a social constructionism perspective, participation and learning go hand-in-hand, where the learner changes as a result of reflection on direct experiences [47, 48]. There were three assessment components: attendance requirement, end-of-year Objective Structured Clinical Examination (OSCE), and a reflective essay. The students were expected to meet the minimal attendance requirement of 80% of all the course sessions. The OSCE was summative, based on pass/fail criteria. The OSCE was multi-specialty and focused on core generic clinical skills and included aspects related to professionalism. The reflective component consisted of a 500-word essay, asking students to relate their experiences in the clinical placements to the concepts taught in the course. The instructions around the reflective essays were purposefully kept to a minimum to avoid restrictions, maximizing the reflection process.

### Research design

This qualitative study was based on a phenomenological research design. Constructivist epistemology comprised the theoretical underpinning of the entailed interpretive qualitative analysis [49]. The analysis followed the six-step approach initially introduced by Braun and Clarke (2006) [50]. This inductive multi-staged methodology is encouraged in qualitative socio-behavioral research [51]. By capitalizing on this participant-focused research design [52], the researchers were able to tap into the participants’ lived experiences. This study was approved by the Institutional Review Board of MBRU (MBRU IRB-2023-175).

### Data retrieval

The dataset analyzed for the purpose of the current study constituted the reflective essays prepared by the students of a single MBBS cohort (*n* = 33) in academic year 2020–2021. The reflective essay is one of the three required assessment components of the respective course. Out of the respective MBBS students, 76% were female. They were of 14 nationalities, 39% were UAE nationals. All submitted essays were included in the study. The participating students were informed that the content of their reflective essays was analyzed for the current research study. To protect the anonymity of the participants, each was assigned a unique identifier, composed of two parts: a serial number (i.e., 01 to 29), followed by “F” for female or “M” for male. For example, the identifier: 21 F, represents participant number 21, who is a female.

### Data analysis

The data analysis commenced after the conclusion of the data retrieval phase. The data analysis was inductive. The analysis process was iterative and based on constructivist epistemology [52]. This was done using a participant-focused, phenomenological approach to thematic analysis by three data analyzers (R.B., F.O., and S.B.H.). Prior to the analysis, they identified personal characteristics that they collectively believed may influence their perceptions in relation to the subject matter. Consistency, regarding the underlying assumptions and theories, was assured throughout the process by one of the data analyzers (F.O.) who has developed expertise in qualitative socio-behavioral research. This interpretative approach is different than standard scientific inquiry and involves the ability to acknowledge and recreate the experiences of the participants. The goal of this approach is to understand and relate to individuals, and their thoughts and ideas, and motives, aspirations, and actions, rather than to find casual explanations. This methodology assumes that we can interpret individuals’ thoughts, emotions, and behaviors by actively listening and understanding what they are saying/ their self-expressions.

The Braun and Clarke six-step framework [50] constituted the adapted qualitative analysis. This multi-phased approach to thematic analysis is widespread and has been widely used in research around health professions’ education [53, 54]. NVivo software version 12.0 plus (QSR International Pty. Ltd., Chadstone, Australia) was used to code the data (i.e., assign labels/ titles to the sub-categories, categories, and themes), and in turn facilitate the categorization of the text fragments highlighted by the data analyzers.

**Fig. 3:**
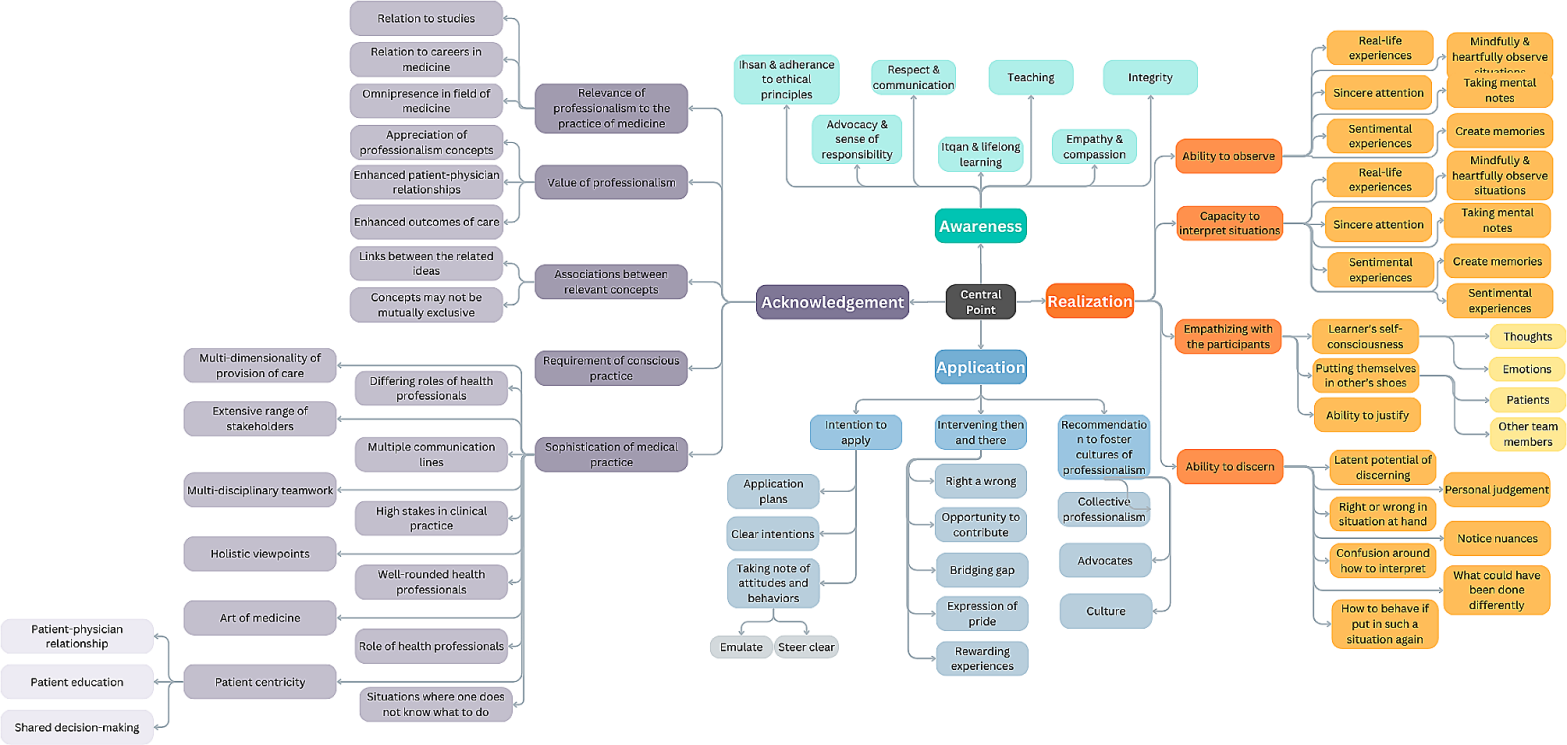
Mind map deployed as a tool to facilitate the qualitative analysis

The analysis process is described by the following steps:

1. Familiarization with the data. The data analyzers (R.B., F.O., and S.B.H.) worked as a group and initially took turns in reading out loud the essays to familiarize themselves with the compiled dataset. They thoroughly reflected upon the content of the de-identified data, and shared any thoughts and ideas that surfaced for them.
2. Generation of initial codes. Text fragments from the essays that related, directly or indirectly, to the overall purpose of the study were extracted. As such, any text segment relating to the participants’ understanding of, and personal experience with professionalism, and their engagement with the content of an innovative curriculum-based professionalism course was tagged. This process continued until data saturation was achieved (i.e., no new information or insights were observed in the datasets). This systematic review led to the creation of categories of text fragments which directed the data analyzers to the next step.
3. Searching for themes. This step was characterized by conducting several rounds of structured reflections. Thus, the different ways by which the identified categories could relate to one another were identified (leading to several potential interconnections).
4. Reviewing themes. The categories were collated to form higher-order themes, according to the linkages that made most sense to the data analyzers (Fig. 3).
5. Defining and naming themes. All the categories and themes were then coded (i.e., given labels/ titles) and defined in the context of the study. This resulted in the study’s conceptual model. Afterwards, as part of this step, a respondent validation was conducted, where a random sample of participants were invited to attend a virtual meeting. The principal investigator (R.B.) provided an explanation of the research questions, the process of qualitative analysis, and the generated conceptual model. After reflecting upon the extent of resonance between their responses to the research questions and the generated conceptual model, the meeting attendees agreed with all the identified codes (i.e., themes and categories), and how the current study’s conceptual model portrays the linkages across those themes and categories.
6. Reporting on findings. The results of the abovementioned steps were reported (i.e., Results section) narratively in alignment with established guidelines, including the Standards for Reporting Qualitative Research (SRQR) [55–58] as part of this step. To further substantiate the findings, two tallies were conducted. First, the data analyzers reported on the number of analyzed text segments within each category, within the recognized themes. If for a single participant, more than one relevant text fragment was put in the same category, they were all collectively considered as one entry. Accordingly, the first tally reflected the number of participants that brought up matters relevant to each of the categories. Second, the data analyzers read all participants’ reflective essays again, but this time to identify and tally the professionalism concepts as per the outline of the curriculum under investigated.

## Results

**Fig. 4:**
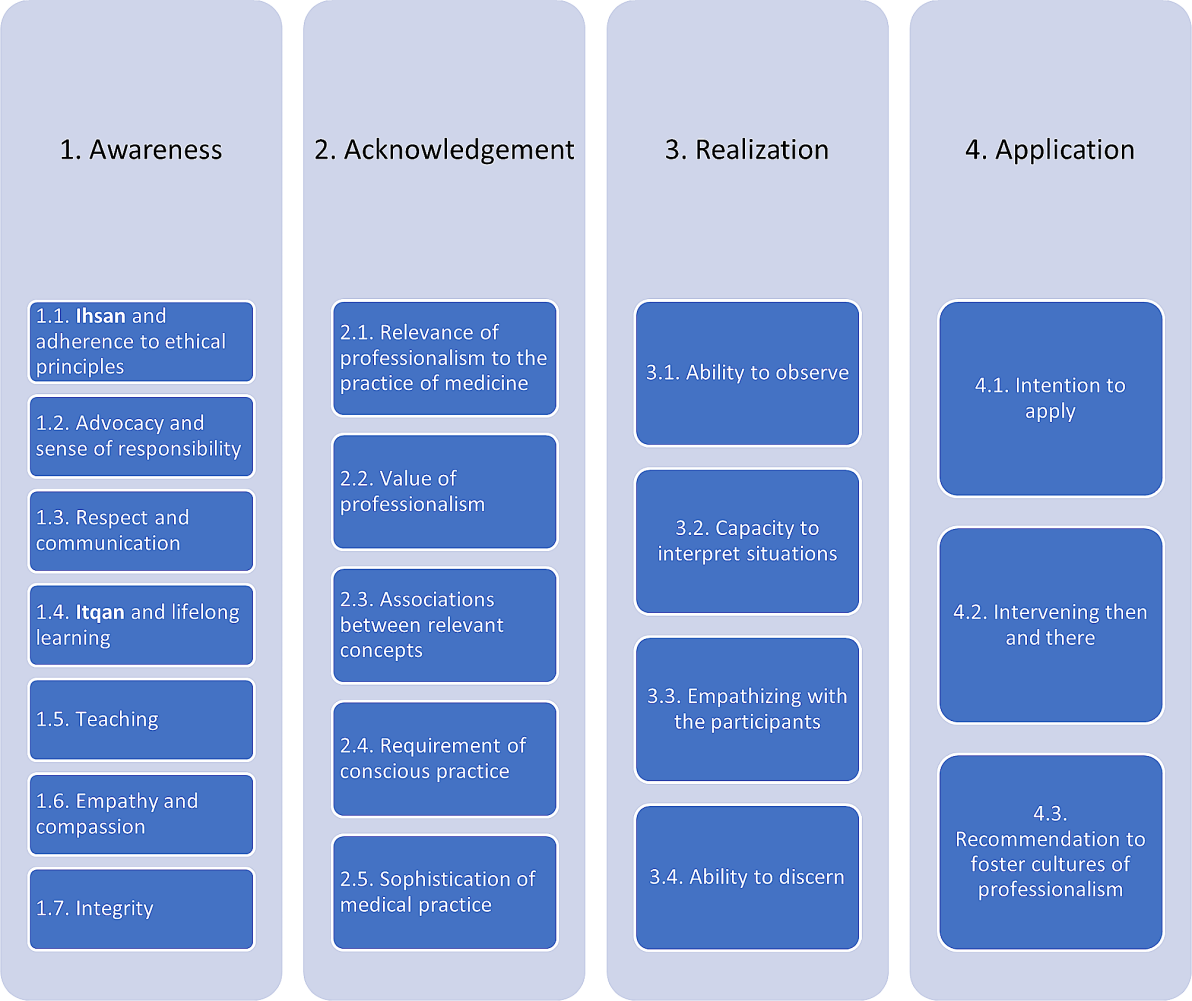
The current study’s conceptual model: *Professionalism Learning Journey*

Out of the 33 enrolled students of the respective MBBS cohort, 29 submitted their reflective essays (the remaining four students were formally exempt from the exercise for differing reasons including study abroad and year repetition). The qualitative analysis led, as per this study’s conceptual model: *Professionalism Learning Journey* (Fig. 4), to four interconnected themes, namely: Awareness, Acknowledgement, Realization, and Application. Within Awareness, seven categories were identified: Ihsan and adherence to ethical principles, Advocacy and sense of responsibility, Respect and communication, Itqan and lifelong learning, Teaching, Empathy and compassion, and Integrity. Within the second theme: Acknowledgement, the following five categories were identified: Relevance of professionalism to the practice of medicine, Value of professionalism, Associations between relevant concepts, Requirement of conscious practice, and Sophistication of medical practice. As for the third theme: Realization, the following four categories were identified: Ability to observe, Capacity to interpret situations, Empathizing with the participants, and Ability to discern. Lastly, within the fourth theme: Application, the following three categories were recognized: Intention to apply, Intervening then and there, and Recommendation to foster cultures of professionalism.

The tally of the count of text fragments belonging to each category showed the distribution, outlined in Table 1.

**Table 1:** Semi-quantitative tally of the output of the participant-focused qualitative analysis

Theme	1.Awareness							2.Acknowledgement					3.Realization				4.Application		
Category	1.1.	1.2.	1.3.	1.4.	1.5.	1.6.	1.7.	2.1.	2.2.	2.3.	2.4.	2.5.	3.1.	3.2.	3.3.	3.4.	4.1.	4.2.	4.3.
**Tally of participants (out of 29)**	3	2	5	4	3	5	3	7	11	8	9	14	13	7	6	14	11	17	17
**Sum of exemplars per theme**	25							49					40				45		

The tally of the count of topics covered in participants’ reflection essays showed the distribution, outlined in Table 2.

**Table 2:** Quantitative tally of professionalism concepts covered

Concept/ Category	1.1.Ihsan and adherence to ethical principles	1.2.Advocacy and sense of responsibility	1.3.Respect and communication	1.4.Itqan and lifelong learning	1.5.Teaching	1.6.Empathy and compassion	1.7.Integrity
**Tally of participants (out of 29)**	8	5	26	8	3	24	11

## Awareness

This theme encapsulated all the text fragments that referred to the participating learners’ personal interpretations of the theoretical knowledge that they had acquired as part of the professionalism course. These text fragments constituted the participating learners’ perceptions of the taught concepts, irrespective of whether, or not, their reflections were congruent with what was taught. When describing the concepts, from their point of view, the participating learners sounded as if it is always a black-and-white subject or situation. Also, the participating learners, when reflecting on the taught concepts, appeared to be referring to professionalism that is exhibited on an individual level.

Although, the adapted analysis approach was purely inductive (as described in the Methods section), the data analyzers chose, for this particular theme only, to superimpose the components of the content of the course under investigation onto what got encapsulated within this theme. Although, the inductive analysis showed that all the taught concepts were mentioned, these were not equally identified in the essays (Table 2), with most reflecting on respect and communication, and empathy and compassion. Hence, to avoid unnecessary confusion and to enable an accurate quantitative tally of taught concepts, the researchers mindfully chose to minorly modify the inductively generated categories of the first theme: Awareness, to align with the course structure.

### Ihsan and adherence to ethical principles

This category covered text fragments where the participating learners’ description of how professionalism in medical practice is at least partly related to experiencing a sense of accountability to a higher presence

5 M: “…The concept of Ihsan stems from the feeling of not only holding oneself accountable for one’s actions towards patients, families, and/ or the organizations we work for, but also to a divine being…”.

14 F: “…The term ‘Ihsan’ holds a deep, spiritual value since it brings together one’s faith and religion, and the intention behind behaving in any particular way which consequentially generates more goodness…”.

### Advocacy and sense of responsibility

This category related to expressions which showed the participating learners’ beliefs that health professionals should protect the ethical principle of equity by advocating for quality health care for all individuals. This category also encapsulated expressions of the participating learners’ beliefs pertaining to health professionals’ responsibility.

26 F: “…As medical professionals, we have a lot of responsibilities that we need to live up to… This experience highlighted important concepts of professionalism including responsibility, altruism, and advocacy. The responsibility of care is based on a cooperative effort among professionals of differing specialties including but not limited to doctors, nurses, pharmacists, laboratory and radiology technicians, and receptionists…”.

28 M: “…Medical professionals are responsible for their patients’ lives, any error can lead to death or disability…”.

### Respect and communication

This category focused on the participating learners emphasis of health professionals’ requirement for respectfully and effectively communicating with colleagues, patients, and their carers.

1 F: “…Physicians must ensure sufficient communication with the patient and the patient’s family, as well as other involved members of the healthcare team…”.

12 F: “…Understanding the importance of communication is a major step towards becoming a professional healthcare worker…”.

### Itqan and lifelong learning

This category brought together all that was expressed by the participating learners in relation to the requirement among health professionals for Continuing Professional Development, and of exhibiting excellence while interacting with others and performing their duties.

13 F: “…Lifelong learning enables the doctors to better manage patients which in turn leads to better outcomes of care…”.

16 F: “…Itqan refers to the attainment of the highest level of quality work, and the correct and complete performance of duties…”.

### Teaching

The participating learners repeatedly alluded to the responsibility of healthcare professionals to teach others (e.g., patients or other healthcare team members, including the future physicians).

13 F: “…just as the physicians teach students, students can also serve as a reminder and source of information for physicians…”.

23 M: “…professionals who are involved in teaching students should acknowledge the importance of teaching, and respect students through actions such as commitment to time, equity, and enthusiasm…”.

### Empathy and compassion

There were plenty of examples of expressions made by the participating learners around the health professionals’ responsibility of understanding and sharing the experiences of whom they are interacting with.

1 F: “…empathy is feeling your patients’ concerns by putting yourself in their shoes, and compassion is taking action towards making a change for the better in your patients’ lives…”.

3 F: “…Being a physician means much more than being a doctor who provides treatments for ailments, it is to take care of the patient with honesty, compassion, and empathy…”.

### Integrity

The last category within this theme related to the participating learners’ impressions about the responsibility of health professionals to remain real and true to themselves while fulfilling their professional duties.

2 F: “…Personal gain on the doctor’s side should never come at the expense of the patient’s health…”.

22 F: “…integrity which demonstrates honesty and transparency in the way that a person conducts themselves in the job…”.

## Acknowledgements

This theme included all the fragments of texts that highlight the participating learners’ reflections on what became apparent to them (in terms of medical professionalism or otherwise) as a direct consequence of their engagement in the respective course and its content.

The participating learners highlighted matters that became apparent to them due to the course. In a sense, the saying that ‘once you see it, you cannot unsee it’ became quite evident to how the participating learners related to the differing aspects of professionalism in medical practice. It was apparent from the fragments of texts that the participating learners started going through an attitudinal shift upon acknowledging the value of the knowledge of the taught concepts.

### Relevance of professionalism to the practice of medicine

The participating learners seemed to recognize that the concepts that they were taught as part of this course are highly related to their medical studies and careers. It was clear to the participating learners that the concepts of professionalism are omnipresent in the field of medicine, where they are widely spread and constantly encountered.

5M: “…If we successfully manage to implement professionalism correctly in our first encounters with patients and with fellow healthcare workers, then we are hopefully paving the way to a fruitful and successful journey in medicine…”

10F: “…These experiences have broadened my appreciation and understanding of the impact of communication in the healthcare setting… Every interaction we have has a direct and irreversible effect on the overall patient care…”

### Value of professionalism

The participating learners repetitively alluded to their appreciation of the professionalism concepts, where they believe that abiding to and exercising those concepts lead to enhanced patient-physician relationships and improved outcomes of care.

1F: “…I now understand and appreciate the importance of the professionalism concepts and their implication in every day medical practice…”

26F: ”…I Understood That Good Communication Is Key To Better Outcomes Of Care. Respecting The Patients And Their Situations Will Result In Trustworthy Relationships…”

### Associations between relevant concepts

At many instances in their reflective essays, the participating learners referred to the links between the inter-connecting ideas of the taught course.

7F: “…Communication is the sturdy basis to apply all other ethical responsibilities of professionalism…”

15M: “…using the tools of connectivity, communication, community, and compassion, you can decipher the hidden philosophy of the field of medicine…”

In some cases, the participating learners reflected upon how the concepts may not be mutually exclusive.

6F: “…To practice Itqan includes practicing Ihsan…”

### Requirement of conscious practice

It was clear to the participating learners that they need to mindfully develop the skills around any one concept.

5M: “...The network of healthcare is vast, and practicing effective communication is a major step towards becoming competent healthcare workers….”

10F: “…I now also appreciate just how significant of a role communication plays in any multi-disciplinary team, and is an area any medical student should regularly practice and get better at…”

12F: “…It is important to start working on our empathy as it is an innate capacity and learned behavior that needs to be developed…”

### Sophistication of medical practice

All the participating learners’ reflections that related to the multi-dimensionality of the provision of care were encapsulated in this category. The participating learners seemed to be cognizant of the differing roles that any one health professional exercises. They were also aware that there is an extensive range of stakeholders with whom health professionals need to engage using multiple communication lines as part of multi-disciplinary teamwork.

4F: “…The case management will be approached in a team-based manner where doctors and other healthcare professionals from different disciplines work together... the patient will play a key role in deciding on the appropriate treatment regimen…”

Moreover, the participating learners understood clearly the ‘high-stakes’ nature of medical practice. Some of the participating learners reflected on the importance of holistic viewpoints when caring for patients and their families. The participating learners frequently alluded to the value of well-rounded health professionals.

19F: “…Holistic care encourages physicians to treat their patient as a whole and not just their diseases or illnesses…”

24M: “…You must always look at your patients as human beings, and not just as a name on a checklist that you need to tick off by the end of the day…”

It was also evident to the participating learners that there is the practice of science and art in medicine. The significance of the role of health professionals was also brought up by the participating learners at several instances.

15M: “…I want to remind people that medicine is a humanitarian field as much as it is a scientific one…”

The participating learners also acknowledged the concept of patient centricity, reflecting upon the patient-physician relationship, educating the patient, and shared decision-making. Interestingly, there were a handful of participating learners who expressed surprise when acknowledging that in the practice of medicine there is a possibility of reaching situations where one does not know what to do, and may experience helplessness.

4F: “…It was a scenario that if I were to be put in, I would not have known how to respond…”

6F: “…Perhaps if I was alone with the patient, I may not have been able to handle a sensitive moment such as this one…”

## Realization

This theme included any depiction of an increase in the participating learners’ capacity to relate their experience in the clinical setting to the course content. The learning experience integral to this course helped the participating learners in realizing their potential to access and reflect upon the hidden curriculum. In the clinical environment, the participating learners realized that what is right or wrong, in terms of professional behavior is relative and highly dependent on the circumstances. This constituted a reality check for the participating learners and seemed to substantially expand their capacity to interpret differing situations. It was apparent from the fragments of texts that the participating learners’ realization of their potential in professionalism came hand-in-hand with the concurrent experiential learning that was happening in the clinical setting (by virtue of the structure of year 4).

### Ability to observe

Many text fragments encapsulated in this category referred to the participating learners’ description of real-life experiences that resonated with the taught concepts. The participating learners were able to mindfully and heartfully observe situations where they were able to pay attention to these experiences. It appeared as if they kept mental notes of what was happening around them. In many instances, the participating learners reported on memorable sentimental experiences.

6F: “…I looked at the doctor, who appeared very calm and composed…I observed how the doctor handled the situation… embodiment of empathy in patient care; I will always keep that in mind…”

9F: “…she regarded the patient’s feelings immediately, although what was actually going on was outside the scope of the consultation… witnessing my senior doctor support her patient is an encounter I will always keep in mind…”

### Capacity to interpret situations

It was clear from the text fragments that the participating learners’ capacity to reflect, and interpret situations and conversations expanded as a result of the learning experience. The text fragments showed how the course increased the participating learners’ ability to learn from experience, acquire new skills, and develop new insights. In many instances, the participating learners were going beyond the obvious in their interpretations.

9F: “…As the topic was exceptionally sensitive, the doctor took her time to emotionally support the patient during her visit, the doctor was very considerate when choosing her words and statements…”

11F: “…I have seen the doctor performing such empathetic acts multiple times and to different patients, because she is a human first and a doctor second…she follows treatment guidelines, and only provides such products when needed and when it is of real benefit to the patient…”

### Empathizing with the participants

The course clearly raised the participating learners’ self-consciousness as they reflected widely on their thoughts and emotions in particular situations. They became better at putting themselves in other’s shoes, not only in relation to the patients but also other clinical team members.

Their capacity to justify why things are the way they are was also raised as a consequence to the learning experience integral to the respective course.

1F: “…I expected her to unknowingly show her annoyance at how things had been unfolding to her patient, for she is only human…”

14F: “…From my experience during clinical rotations, I began to understand the combined effect of a myriad of factors such as stress, potentiality human error, and overbooked appointments which can be mistakenly perceived by the receiver of care as a ‘bad doctor’…”

### Ability to discern

The respective course seemed to support the participating learners in exploring and in turn realizing what seemed like a latent potential of discerning, based on their own personal judgement (in the situation at hand), between what is right or wrong. The participating learners seemed to notice the nuances of the different encounters. In some instances, the participating learners described instances where, then and there, they were not sure how to interpret the situation. In hindsight (as part of the reflective exercise), however, they pointed out what could have been done differently, and/ or how they would want to behave if they are put in such a situation again.

7F: “…his behaviors inspired me...I left the room feeling certain that I realized how I want to show-up in my future practice…”

15M: “…I see many doctors practicing medicine like advanced computing robots rather than compassionate humans…”

16F: “…some of the physicians throw sarcastic comments about their patients behind the scenes. We will not accept such a behavior if those receiving the care are members of our families…”

## Application

The last theme of the generated conceptual model referred to expressions from the participating learners about how they put into practice what has been acquired, as well as giving practical recommendations.

### Intention to apply

This category encapsulated all text fragments that showed that the participating learners were taking note of the attitudes and behaviors that they were observing to either emulate or avoid (to get better outcomes) in their own future practice. They expressed their plans to apply what they had been learning from observation. Their intentions were clear.

13F: “…As I go on through the course of my career, it is my hope to continually exhibit respect and good communication towards my patients, colleagues, and people generally… I plan to be open to constantly learning as this will enable me to be a better physician and a better person…”

15M: “…I will continue to include the concepts covered in our professionalism course into my daily practice, regardless of how challenging it may be…”

### Intervening then and there

Interestingly, the participating learners reported on times where they appear to have attempted to ‘right a wrong’. They reflected upon instances where they saw an opportunity to contribute, and they acted in a way to bridge the gap.

3F: “…I tried my best to calm the child down and to reassure her that the doctor will try his best not to hurt her and that she is brave…”

8M: “…I tried to be mindful of the patient’s body language… I tried to acknowledge the distress… I tried to accommodate... As a result, the patient became in a much better mood…”

They expressed pride, and described the situations that they rectified through intentional acts of professionalism as ‘rewarding learning experiences’.

10F: “…all I did was lend a listening ear and some kind words, and I could tell that how I behaved meant so much to her… and that alone managed to put a smile on her face… I was also left feeling very accomplished…”

### Recommendation to foster cultures of professionalism

The last category in this theme referred to participating learners’ reflections on means to advance collective professionalism. The participating learners were clearly advocating for what physicians need to do. The participating learners kept referring to ideas that can be implemented to promote cultures of professionalism.

23M: “…I encourage all medical professionals, at all levels of training, to reflect on the importance of communication in medical practice, and to intervene to address gaps in communication when they arise...improved communication across the board will lead to a better healthcare system…”

26F: “…I would like to remind myself and all the people working in the medical sector to be professional with their patients and surroundings by first and foremost demonstrating empathy, engaging in shared decision making, and abiding to behavioral and medical ethics principles…”

## Discussion

The professionalism-training course, reported upon in the current study, was positively received by the learners. The current study offers a deep understanding of medical students’ perceptions of medical professionalism. Reflection is called for in medical education, especially in relation to bioethics and professionalism, and is becoming more widespread [12, 59]. There are several medical schools around the world that have effectively incorporated reflection in their formal curriculum and/ or co-curricular programs. Reflection teaches medical students to step back and analyze their learning experiences [59]. For example, Loyola University Chicago Stritch School of Medicine incorporated reflection through all four years of their undergraduate medical curriculum, including bioethics education and professional development efforts [59]: (1) three-year longitudinal clinical skills course Patient Centered Medicine (PCM), (2) co-curricular Bioethics and Professionalism Honors Program, and (3) in their Physician’s Vocation Program (PVP).

It is established that professional values, attitudes, and behaviors are intrinsic to all medical practice, yet (as previously mentioned) professionalism remains one of the most intangible subjects. It is also known to be among the most difficult to integrate into medical curricula in an explicit manner [17, 60]. The twenty-first century raises challenges not only to adapting the means of training professionalism to changing societal values but also for instilling skills of ongoing self-directed learning. The *Professionalism Learning Journey*, in the current study, appeared (based on the inductive qualitative analysis) to cover 4 interlinked stages. Accordingly, the learning journey starts with increasing the knowledge base about professionalism concepts followed by acknowledgement of the value of what have been acquired and then realization of capacity to learn from experience and lastly to put into practice what has been acquired. These stages do not seem to be linear; it is an iterative process that the learners appear to go through as they develop their contextualized medical professionalism competencies. Similarly, another qualitative study explored undergraduate medical students’ understanding of medical professionalism using Team-based Learning (TBL) at the two campuses of Royal College of Surgeons Ireland (RCSI)- Dublin and Bahrain. The thematic analysis generated four unique themes: incoming professional attitudes (i.e., a detached individual from the experiences and perspectives of others), transformative experiences (i.e., significant expansion in learners’ behaviors and attitudes), sociological understanding of professionalism (i.e., segregation of responsibilities based on the level of contribution), and new professional identity formation [2]. It is suggested that the core elements of these themes can be integrated into the teaching of professionalism to prepare fit-to-practice future doctors.

The current study introduces a conceptual model that, in conjunction with the professionalism-training course curriculum, can be leveraged by other medical schools (characterized by similar contexts) to replicate the learning experience. The true practical value of this research work is actually in the transferability of its findings to other similar contexts. The output of this research work can serve as a template for other competencies and other schools. Numerically, the maximum number of exemplars turned out to be in the “acknowledgement” theme followed by the “application” theme and then the “realization” theme and lastly the “awareness” theme. One way of interpreting this is that acknowledging the value of professionalism and its training constituted a turning point (i.e., the start of the previously explained *attitudinal shift*) in the learners’ trajectory. The learners seemed to cherish the experiences associated with the applicability of the content that they had acquired through the respective course. Similar to the *attitudinal shift* that emerged from the current study’s analysis, Guraya et al. (2023) pinpointed a step in the professionalism learning trajectory, namely: *transformative experience*. In this step, the learners appeared to experience a shift in their understanding of the subject matter, which resulted in a significant expansion in their behaviors and attitudes [2].

The learners’ reflections on the sophistication of the medical practice, and the segments of the text that show the students’ ability to discern and take note of nuances stood out the most to the current study’s researchers. The text fragments encapsulated in those categories show the importance of role models, where both positive and negative behaviors were observed by the learners. A study investigated the perception of 386 Australian undergraduate medical students in 2010 about medical television dramas in relation to professional identity development. This qualitative analysis showed 3 dichotomous themes, ‘cure-care’, ‘work-leisure’, and ‘clinical-administration’. The corresponding interpretation showed that participating students had carefully thought about how the representations had meaning for them as developing professionals, from both positive and negative role modelling [61]. Irrespective of the context of observation, mentoring and reflection are important to ensure appropriate learning is achieved. Ideally, the training assessment needs to leverage multiple tools, like the configuration integral to the professionalism-training course investigated in the current study. There is an evident need for robust systems to ensure understanding, learning, and valuing professionalism within medical training [22, 62]. Although all the seven professionalism concepts, taught in the training reported upon in the current study, were given equal importance/ weight in the course delivery, “respect and communication” followed by “empathy and compassion” were brought-up and reflected upon the most in the reflective essays. These topics seem to resonate the most with the learners. This may be because of the stage at which the learners are at and/ or the culture in which they are obtaining their training.

The course reported upon in the current study is an example of starting with the end in mind, where the competencies which were developed (as part of the respective course) were based on what was previously agreed to be of relevance and value in the Gulf Region. Professionalism reflects societal values, and hence, an institutional definition needs to be agreed upon. Learning professionalism in the early years is not enough; it must be supported in the workplace. It is important to find ways to systematically develop professionalism through structured training across the medical education continuum and not solely as part of undergraduate programs [63, 64]. The corresponding initiatives need to take the form of training processes, nurturing professionalism in stages (ideally, within longitudinal horizontally and vertically integrated curricula), and to be complemented with effective, context sensitive assessments of professional identity formation [4]. Also, it would be important to work towards developing, in a standardized manner, the teaching professionalism competencies of the faculty who will deliver the training. Along these lines, Steinert et al. (2005) reported on a comprehensive faculty development program aimed at developing competencies for teaching and assessing professionalism. The program consisted of think tanks to promote buy-in and the development of a common ground, and a series of workshops to convey core content, examine teaching and assessment methods and strategies, and promote reflection and self-awareness [13].

The assessment of the training reported upon in the current study appeared comprehensive, mirroring the respective curriculum intent, yet it does not guarantee the learners’ professional behaviors in the clinical context. Relevantly, it is established that a valid and reliable assessment of core professionalism attributes of undergraduate medical students is essential in ensuring future physicians behave in ways that best serve society. Sattar and Yusoff (2020) suggest Entrustable Professional Activities (EPAs) as an assessment method in competency-based education that could drive professionalism learning [65]. Within this context, they introduce the professional progress pyramid: Attributes, Baseline, and Core (ABC) of EPAs. The core elements of professionalism identified include: Altruism, Accountability, Excellence, Duty, Honor and Integrity, and Respect for others [65]. It was suggested that professional identity formation relates to constructs such as professionalism, leadership, and resilience [66]. Hence, the need to identify a method to quantitatively assess these key areas has been repetitively alluded to in the literature. PILLAR, introduced by Ryan et al. (2023), presents one potential method to do so [66].

To prevent minor discrepancies between the categories (and codes) surfacing from the inductive analysis (around the first theme, namely: Awareness) and the agreed upon professionalism concepts, the data analyzers chose to superimpose the structure of the course (i.e., the 7 professionalism components) onto the respective output of analysis. Such purposeful methodological refinements are known to fine-tune the analytical intentions and in turn strengthen the study’s trustworthiness [51, 67].

This study has a few limitations. Including a single cohort from one medical university enabled the development of in-depth insights. However, the findings are only transferable to institutions which are contextually like that in which the current study took place. Moreover, the current study cohort was 76% female, and there is some evidence of gender differences in perception of the importance of professionalism [68]. It would be valuable for future studies to investigate the perception of learners of learning and teaching interventions, similar to the one investigated in the current study (aimed at developing professionalism), across several medical schools (with attention to greater diversity). In addition, the qualitative narrative data, combined with the phenomenological participant-focused approach in the current study, allowed the researchers to tap into the students’ lived experiences which holds significant value, in terms of the research findings. However, in terms of reliability of the methodology, it would be worthwhile for upcoming studies to deploy a mixed methods approach to research that systematically integrates qualitative with quantitative findings. Moreover, this study enabled the development of an impression of the efficacy of the professionalism-training course but not really its effectiveness. There is also the social desirability bias that might have affected the validity of the research findings, given that the students likely chose to document the reflections that they believe are more appropriate. It would be interesting for future studies to demonstrate whether, or not, there are lasting changes in the attitudes and behaviours of learners in the medical profession (and in turn the outcomes of care) that occur as a result of such a professionalism-training. It may also be worth investigating social and cultural variations in the willingness of individuals to disclose personal struggles which may have an impact on fitness to practice and the extent to which this could be addressed through professionalism-training. Other areas to explore include the attitudes towards and role of social media. While it may be viewed positively by medical students and trainees, its excessive use can also be associated with poor health behaviours, anxiety, depression, and social isolation [69]. In terms of professional identity, social media can also be viewed as risky, and contribute to a dichotomy in which the professional identity is seen as a mask, contributing potentially to some resentment about having to hide part of oneself [15].

## Conclusion

This study showed that integrating a professionalism-training course into medical curricula increases the learners’ awareness about professionalism. It enables them to value the subject matter, realize the sophistication of its application, and put into practice the principles on an individual basis and collectively. This is especially true when the learning and teaching intervention is anchored in constructivism experiential learning theory and designed to foster self-directed learning. This study provides support for further developing and implementing contextualized learning opportunities for fostering professionalism among future healthcare workers.

### Electronic supplementary material

Below is the link to the electronic supplementary material.

Supplementary Material 1

## Data Availability

Data is provided within the manuscript or supplementary information files.
