# Supplementary material for: Professionalism-training in undergraduate medical education in a multi-cultural, multi-ethnic setting in the Gulf Region: an exploration of reflective essays
Source: BMC Med Educ. 2024 Feb 6;24:117. doi: 10.1186/s12909-024-05103-z (PMC10848390; doi:10.1186/s12909-024-05103-z)
Supplement: Supplementary file 1 — Supplementary Material 1 [file 12909_2024_5103_MOESM1_ESM.docx]

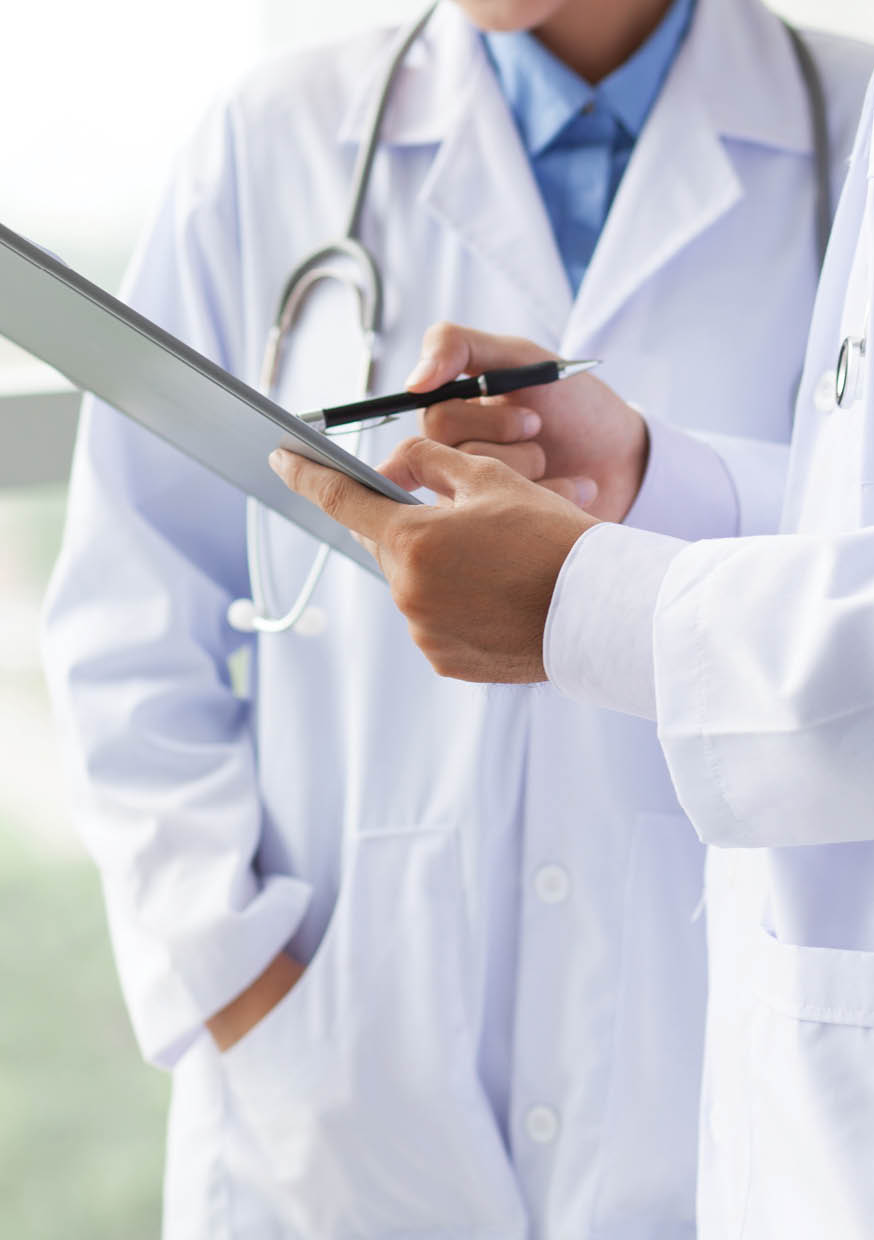

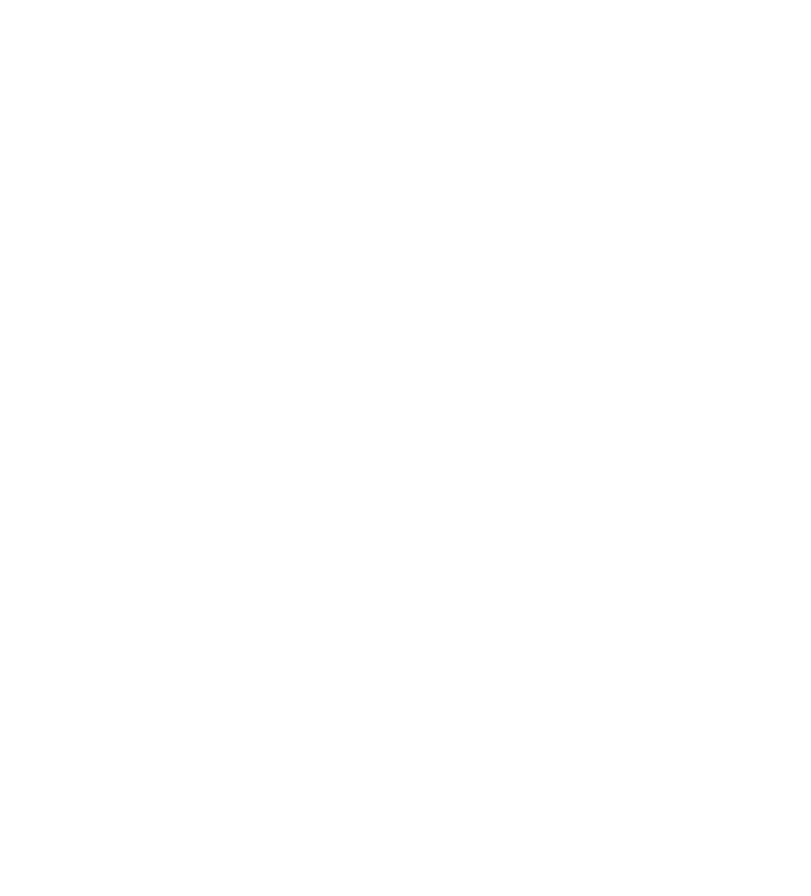


**Professionalism Training**

COURSE GUIDE

**YEAR 4**

**ACADEMIC YEAR 2019-2020**

Contents

[MBRU Faculty & Administrative Staff 1](#_Toc19103327)

[Course Information 2](#_Toc19103328)

[General Information 3](#_Toc19103329)

[Assessment 3](#_Toc19103330)

[Learning Activities 4](#_Toc19103331)

[MBRU Days - Course Schedule 4](#_Toc19103332)

# MBRU Faculty & Administrative Staff

| **Name** | **Role** | **Email** |
| --- | --- | --- |
| **Course Director** |  |  |
| Dr. Rasha Buhumaid | Assistant Professor- Emergency Medicine | Rasha.Buhumaid@mbru.ac.ae |
| **Course Co-Director** |  |  |
| Professor Samuel Ho | Chair- Clinical Sciences | Samuel.Ho@mbru.ac.ae |
| **Faculty** |  |  |
| Professor Mutairu Ezimokai | Provost | Mutairu.Ezimokhai@mbru.ac.ae |
| Professor David Hickey | Professor of surgery | David.Hickey@mbru.ac.ae |
| Dr. Adrian Stanley | Phase III Director | Adrian.Stanley@mbru.ac.ae |
| Dr. Lisa Jackson | Associate Professor - Family Medicine | Lisa.Jackson@mbru.ac.ae |
| Dr. Hany Swidan | Assistant Professor - Family Medicine | Hany.Swidan@mbru.ac.ae |
| Dr. Hanan Al Suwaidi | Assistant Professor - Family Medicine | Hanan.Alsuwaidi@mbru.ac.ae |
| Mr. Hashim Elsayed | Coordinator | Hashim.Ahmed@mbru.ac.ae |
| Ms. Sabah Kiran | Coordinator | Sabah.kiran@mbru.ac.ae |
| Mr. Glorio Almeida | Coordinator | Glorio.Almeida@mbru.ac.ae |

# Course Information

**Synopsis**

This course consists of 9 hours of core teaching during the clinical rotations of Year 4 of the Bachelor of Medicine and Bachelor of Surgery program (MBBS). It provides students with an introduction to the concept of professionalism in medicine. The course will focus on the core principles and attributes expected from physicians, emphasizing on the concepts that are unique to the medical practice in the middle east. The course will help students build a foundation of professional medical practices that will be used throughout their career.

By the end of the course, the students shall be able to:

- Explain the following concepts of professionalism in medicine:
- Ihsan and adherence to ethical practice
- Advocacy and sense of responsibility
- Respect and communication
- Itqan and lifelong learning
- Teaching
- Empathy and compassion
- Integrity
- Acquire skills and attitudes to enhance professionalism
- Define the roles of physicians, teams, the healthcare system, and the external environment in supporting professionalism

# General Information

**Learning Management System (LMS):**

The course description will be uploaded in LMS. Prior to entry into the classroom, students should check the LMS for information submitted each week during the course: schedule, assessments, reading materials, and video sites (when/if available).

**Reference:**

**Journal Articles:**

- Abdel-Razig S., Ibrahim H., Alameri H., et al. Creating a Framework for Medical Professionalism: An Initial Consensus Statement from an Arab Nation. Journal of Graduate Medical Education. 2016;8(2):165–172. doi:10.4300/JGME-D-15-00310.1
- American Board of Internal Medicine Foundation, American College of Physicians-ASIM Foundation, European Federation of Internal Medicine. Medical professionalism in the new millennium: a physician charter. Ann Intern Med 2002;136:243-246
- Husser, W.C. Medical professionalism in the new millennium: A physician charter. Journal of the American College of Surgeons. 2003; 196 (1),115-118

**Books in Al Maktoum Library**

- Levinson W., Ginsburg S., Hafferty F., Lucey C. Understanding Medical Professionalism.
- Purtilo R., Haddad M. Health Care Professional and Patient Interaction.
- Makely S. Professionalism in Health Care. A primer for career success.

**Attendance:**

All students should attend all classes. Students, who miss more than 20% of the class sessions, will be automatically dropped from the course. Students are required to come on time to each session, as tardiness is not acceptable.

**Plagiarism:**

The University has established strong policies against plagiarism. In submitting your assignments, plagiarism will not be tolerated, and a failing grade will be incurred. Make sure to read the full text given to you in Student Handbook

***Note:***

*This guide is subject to minor changes. The student will be informed of any changes in the course content, re-scheduling of lectures or tutorials, or any other announcements through the LMS.*

# Assessment

This longitudinal course has three components of assessment: attendance requirement, reflection essay, and final OSCE. The OSCEs will be scored and applied to the clerkship rotation courses.

| **Assessment components** | **Timescale** | **Percentage of overall grade** | **Course outcomes** |
| --- | --- | --- | --- |
| Attendance | Each session | Must have achieved  attendance goal of 80% | Pass/Fail |
| Reflection essay | End of year | Formative | Formative feedback |
| OSCE  Must demonstrate skills related to professionalism | End of year | 75% | Pass/Fail |

# Learning Activities

**MBRU structured learning sessions**

All students in the clerkship rotations will gather for structured learning sessions on Thursdays on the main campus. The sessions will consist of specific clerkship related disciplines (Internal Medicine, Pediatrics, Family Medicine, Behavioral Medicine, and Surgery) and longitudinal topics that are spread over the school year. These will include formal didactic sessions, structured problem-based learning sessions, and student presentations, tutorials, and seminars. The topics related to Professionalism correspond to the core curriculum listed below.

These sessions will be conducted by assigned MBRU faculty. Questions for your final OSCE exam will be derived from the required resources listed below for each topic in the core curriculum.

**On-line tutorials**

Some sessions may require students to complete on-line tutorials or watch videos. Prior to the scheduled teaching sessions, review LMS to determine if any preparation is required.

**Teaching sessions**

- Students complete the assigned online tutorials (if specified on LMS)
- Session Review - essential points of the core topic will be reviewed and major teaching points emphasized. The session will include presentation, case-based discussion and/or role-play.

#

# MBRU Days - Course Schedule

**Sessions**

| **Date** | **Session** | **Timing** | **Faculty** |
| --- | --- | --- | --- |
| 10 OCT 2019 | Introduction to Professionalism | 12.00-13.00Hrs | Professor Samuel Ho/ Dr. Rasha Buhumaid |
| 5 DEC 2019 | Concept of Empathy and Compassion | 14.00-15.00Hrs | Professor Samuel Ho/ Professor David Hickey/ Dr. Rasha Buhumaid |
| 19 Dec 2019 | Concept of Integrity – Workplace conflicts of interest | 12.00-13.00Hrs | Professor Samuel Ho/ Professor David Hickey/ Dr. Rasha Buhumaid |
| 16 Jan 2020 | Concept of Ihsan and adherence to ethical practice | 12.00-13.00Hrs | Professor Mutairu Ezimokhai/ Dr. Rasha Buhumaid |
| 23 Jan 2020 | Concept of Itqan (excellence, going beyond) | 12.00-13.00Hrs | Professor Samuel Ho/ Dr. Rasha Buhumaid |
| 6 Feb 2020 | Concept of Teaching | 12.00-13.00Hrs | Dr. Adrian Stanley/ Dr. Rasha Buhumaid |
| 27 Feb 2020 | Concept of Respect and Communication | 14.00-16.00Hrs | Dr. Lisa Jackson/ Dr. Hany Swidan/ Dr. Rasha Buhumaid |
| 9 Apr 2020 | Concept of Advocacy and Responsibility | 14.00-15.00Hrs | Dr. Adrian Stanley/ Dr. Hanan Al Suwaidi |

|  | | | | | |
| --- | --- | --- | --- | --- | --- |
| ***Activity***  ***Session 1***  **Introduction to Professionalism** | ***Faculty:***  Professor Samuel Ho, Dr. Rasha Buhumaid | ***Mode:***  Tutorial, including small group discussion or role plays | ***Date:***  October 10, 2019 | ***Time:***  12.00 – 13.00Hrs | ***Venue:***  LH 9+10 (RW 3rd) |
| **Learning Objectives** | **Upon completion of the case, the student should be able to:**   - Explain the concepts of professionalism in medicine. - Outline steps to learn skills and attitudes to enhance professionalism in each specialty. - Reflect on how you will be influenced by role modeling on the wards and clinics. | | | | |
|  | | | | | |
| ***Activity***  ***Session 2***  **Concept of Empathy and Compassion** | ***Faculty***  Professor Samuel Ho, Professor David Hickey, Dr. Rasha Buhumaid | ***Mode:***  Tutorial, including small group discussion or role plays | ***Date:***  December 5, 2019 | ***Time:***  14.00 – 15.00Hrs | ***Venue:***  LH 9+10 (RW 3rd) |
| **Learning Objectives** | **Upon completion of the case, the student should be able to:**   - Explain the concepts of compassion and empathy. - Understand the importance of empathy and compassion in the medical field. - Outline steps to learn skills and attitudes to enhance compassion and empathy. - Reflect on how it would be like to be a cancer patient or a critically ill patient and your physician’s impact on your feelings. | | | | |
|  | | | | | |
| ***Activity***  ***Session 3***  **Concept of Integrity – Workplace conflicts of interest** | ***Faculty***  Professor Samuel Ho, Professor David Hickey, Dr. Rasha Buhumaid | ***Mode:***  Tutorial, including small group discussion or role plays | ***Date:***  December 19, 2019 | ***Time:***  12.00 – 13.00Hrs | ***Venue:***  LH 9+10 (RW 3rd) |
| **Learning Objectives** | **Upon completion of the case, the student should be able to:**   - Explain different conflicts of interest that can occur in the workplace and how this affects physician behavior. - Reflect on what it means to have integrity. - Outline the “debate” about the pros and cons of working with big pharma in terms of conflicts of interest and conduct | | | | |
|  | | | | | |
| ***Activity***  ***Session 4***  **Concept of Ihsan and adherence to ethical practice** | ***Faculty***  Professor Mutairu Ezimokhai  Dr. Rasha Buhumaid | ***Mode:***  Tutorial, including small group discussion or role plays | ***Date:***  January 16,2020 | ***Time:***  12.00 – 13.00Hrs | ***Venue:***  LH 9+10 (RW 3rd) |
| **Learning Objectives** | **Upon completion of the case, the student should be able to:**   - List features of the concept of Ihsan. - Discuss meaning of Ihsan in everyday medical practice. | | | | |
| ***Activity***  ***Session 5***  **Concept of Itqan** | ***Faculty***  Professor Samuel Ho  Dr. Rasha Buhumaid | ***Mode:***  Tutorial, including small group discussion or role plays | ***Date:***  January 23, 2020 | ***Time:***  14.00 – 15.00Hrs | ***Venue:***  LH 9+10 (RW 3rd) |
| **Learning Objectives** | **Upon completion of the case, the student should be able to:**   - List features of the concept of Itqan. - Discuss meaning of the expert in everyday medical practice. - List effective methods needed to develop expertise. | | | | |
|  | | | | | |
| ***Activity***  ***Session 6***  **Concept of Teaching** | ***Faculty***  Dr Adrian Stanley  Dr. Rasha Buhumaid | ***Mode:***  Tutorial, including small group discussion or role plays | ***Date:***  February 6,2020 | ***Time:***  12.00 – 13.00Hrs | ***Venue:***  LH 9+10 (RW 3rd) |
| **Learning Objectives** | **Upon completion of the case, the student should be able to:**   - List the attributes related to Teaching and education in your role as a physician - List criteria indicating an individual’s readiness for teaching and learning. - List three techniques needed for your practice as a physician to continue learning. | | | | |
|  | | | | | |
| ***Activity***  ***Session 7***  **Concept of Respect and Communication** | ***Faculty***  Dr. Lisa Jackson  Dr. Hany Swidan  Dr. Rasha Buhumaid | ***Mode:***  Tutorial, including small group discussion or role plays | ***Date:***  February 27, 2020 | ***Time:***  14.00 – 16.00Hrs | ***Venue:***  LH 9+10 (RW 3rd) |
| **Learning Objectives** | **Upon completion of the case, the student should be able to:**   - Describe the various situations in which professionals must communicate with one another. - Explain the importance of treating fellow health care workers at all levels equally and with respect. - Practice interprofessional scenarios including telephone consultations for the purpose of seeking advice on the management of patients. - Describe what needs to be in an interprofessional letter or discharge summary. - Practice a scenario in which the student may be challenged by a junior individual, or the student may challenge a more senior individual about clinical management. | | | | |
|  | | | | | |
| ***Activity***  ***Session 8***  **Concept of Advocacy and Responsibility** | ***Faculty***  Dr. Adrian Stanley  Dr. Hanan Al Suwaidi | ***Mode:***  Tutorial, including small group discussion or role plays | ***Date:***  April 9, 2020 | ***Time:***  14.00 – 15.00Hrs | ***Venue:***  LH 9+10 (RW 3rd) |
| **Learning Objectives** | **Upon completion of the case, the student should be able to:**   - Explain why advocacy is important and circumstances where this relates to the physician’s role in community and societies. - Discuss the concept of responsibility as your role as a physician related to your community. - List three actions that physicians can take related to advocacy and responsibility related to their communities. | | | | |
